# Impact of Unplanned Intra-Operative Conversions on Outcomes in Minimally Invasive Pancreatoduodenectomy

**DOI:** 10.1007/s00268-023-07114-1

**Published:** 2023-07-12

**Authors:** Monish Karunakaran, Matthew Marshall-Webb, Shahid Ullah, Savio George Barreto

**Affiliations:** 1 Department of Surgical Gastroenterology, Asian Institute of Gastroenterology, Hyderabad, India; 2 College of Medicine and Public Health, Flinders University, Bedford Park, South Australia Australia; 3 Division of Surgery and Peri-Operative Medicine, Flinders Medical Center, Bedford Park, Adelaide, South Australia 5042 Australia

## Abstract

**Background:**

Minimally-invasive pancreatoduodenectomy (MIPD) is fraught with the risk of complication-related deaths (LEOPARD-2), a significant volume-outcome relationship and a long learning curve. With rates of conversion for MIPD approaching 40%, the impact of these on overall patient outcomes, especially, when unplanned, are yet to be fully elucidated. This study aimed to compare peri-operative outcomes of (*unplanned*) *converted* MIPD against both *successfully completed* MIPD and upfront *open* PD.

**Methods:**

A systematic review of major reference databases was undertaken. The primary outcome of interest was 30-day mortality. Newcastle–Ottawa scale was used to judge the quality of the studies. Meta-analysis was performed using pooled estimates, derived using random effects model.

**Results:**

Six studies involving 20,267 patients were included in the review. Pooled analysis demonstrated (*unplanned*) *converted* MIPD were associated with an increased 30-day (RR 2.83, CI 1.62- 4.93, *p* = 0.0002, *I*^*2*^ = 0%) and 90-day (RR 1.81, CI 1.16- 2.82, *p* = 0.009, *I*^*2*^ = 28%) mortality and overall morbidity (RR 1.41, CI 1.09; 1.82, *p* = 0.0087, *I*^*2*^ = 82%) compared to *successfully completed* MIPD. Patients undergoing (*unplanned*) *converted* MIPD experienced significantly higher 30-day mortality (RR 3.97, CI 2.07; 7.65, *p* < 0.0001,* I*^*2*^ = 0%), pancreatic fistula (RR 1.65, CI 1.22- 2.23, *p* = 0.001, *I*^*2*^ = 0%) and re-exploration rates (RR 1.96, CI 1.17- 3.28,* p* = 0.01, *I*^*2*^ = 37%) compared upfront *open* PD.

**Conclusions:**

Patient outcomes are significantly compromised following *unplanned intraoperative conversions* of MIPD when compared to *successfully completed* MIPD and upfront *open* PD. These findings stress the need for objective evidence-based guidelines for patient selection for MIPD.

**Supplementary Information:**

The online version contains supplementary material available at 10.1007/s00268-023-07114-1.

## Introduction

The adoption of MIPD into the pancreatic surgical armamentarium has been cautious owing to an appreciation of the technical challenges, high morbidity, pronounced volume-outcome relationship and long learning curves [1–3]. The initial randomized controlled data supporting feasibility of MIPD were from high-volume centres [4, 5] with considerable experience in minimally-invasive surgery. The largest study (MITG-P-CPAM) [6] has shown a marginal, though statistically significant, benefit in terms of post-operative length of hospitalization (15 vs. 16 days). In general, studies comparing MIPD to open PD [7, 8] demonstrate longer operative duration for MIPD with a reduced intraoperative blood loss and comparable post-operative morbidity and mortality. The Dutch multi-centre LEOPARD-2 trial [9] was the first ‘real world’ data to emerge in MIPD. The trial was prematurely terminated owing to increased complication-related 90-day mortality in the MIPD arm (10% vs. 2%) on interim analysis. It paved the way for introspection around the role of MIPD and the need for a systematic approach to the training of surgeons, as well as the uptake of MIPD in a controlled environment to obviate the risk of inadvertent harm to patients.

Not all PDs are equal [10]! MIPDs are fraught with an additional layer of complexity when compared to open PD, namely, the risk of conversion. The rates of this event have been reported to approach 40% [11]. Despite a previous nationwide training programme in LPD [12], the conversion rate during the LEOPARD-2 trial was 20% [9]. Experience from minimally-invasive colorectal [13] and liver [14] resections suggest that an unplanned intraoperative conversion not only nullifies the benefits afforded by minimally-invasive surgery, but may also worsen surgical outcomes. The impact of unplanned intraoperative conversions during MIPD on overall patient outcomes is yet to be fully elucidated [15]. This study aimed to compare peri-operative outcomes of (*unplanned*) *converted* MIPD versus *successfully completed* MIPD and upfront *open* PD.

## Study methodology

### Search strategy

Major databases (Medline, Google Scholar and Cochrane Library) were comprehensively searched to identify all relevant studies published between January 2000 and December 2022 using the MeSH keywords provided in *Supplementary table* 1.

**Fig. 1:**
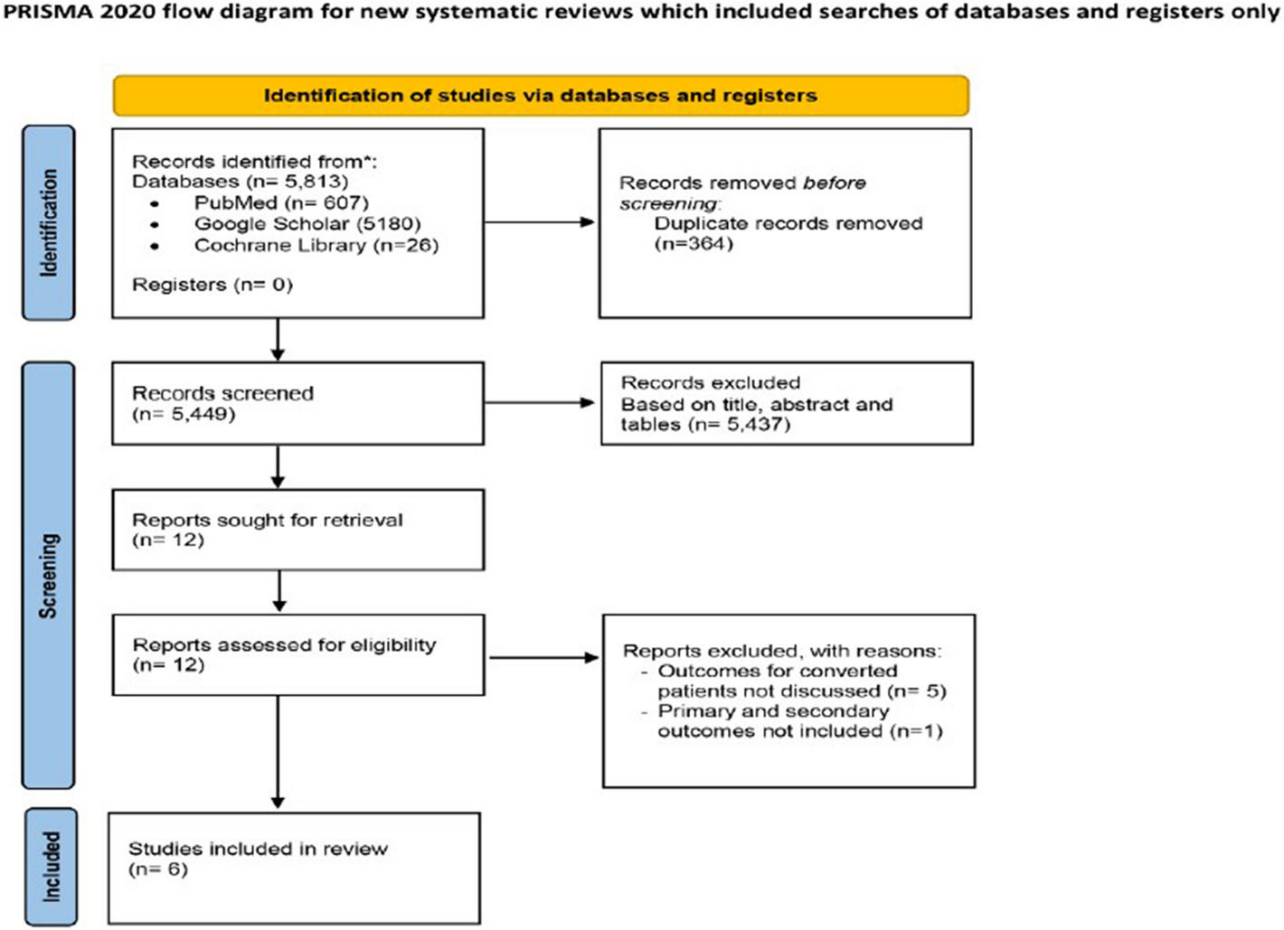
PRISMA diagram

The review was registered with PROSPERO (CRD42022355044) and performed in strict adherence to the PRISMA 2020 guidelines [16] (Fig. 1).

### Inclusion Criteria

Studies fulfilling the following PICOS criteria were deemed eligible for inclusion in the systematic review:

- P (Population): Patients undergoing MIPD (LPD or RPD)
- I (Intervention of interest): Intra-operative Conversion ((*unplanned*) *converted* MIPD). Conversion was defined as any resection starting with a laparoscopic or robotic approach, but requiring either laparotomy or hand assistance for reasons other than trocar placement or specimen extraction
- C (Comparator):
  - Comparator I: successfully completed MIPD
  - Comparator II: upfront *open* PD
- O (Outcomes):reported only by Beane et
  - Primary outcome: 30-day mortality
  - Secondary Outcomes: Pancreas-specific complications [17] including CR-POPF [17] (16), DGE [18], PPH [19], overall and major (≥ CD grade 3) [20] morbidity, re-exploration, length of stay (LoS) and re-admissions.
- S (Study design): Observational, comparative studies

### Exclusion criteria

- Studies without a comparative analysis
- Hybrid PD
- Outcomes of interest not reported
- Studies including extended resections (extended PD and/or extended total pancreatectomy)
- Studies on palliative treatments
- Inability to extract relevant data from published results
- Non-English language studies

### Data extraction and quality assessment

Two authors (MK and MMW) independently extracted relevant data from the screened full-text articles according to a standardized Cochrane data extraction sheet [21], which included the following: Name of the first author, year of publication, sample size, baseline demographic characteristics (including age, sex, BMI), ASA grade, intra-operative characteristics including type of MIPD- laparoscopic or robotic or hybrid, operative duration, blood loss, transfusion requirements and markers of oncologic adequacy including *R0* resection rates and lymph nodal harvest, post-operative outcomes such as CR-POPF, DGE, PPH, overall and major (≥ CD 3) complications, medical complications including cardiac (myocardial infarction, cardiac arrest), pulmonary (re-intubation rates, need for ventilator support > 48 h, pneumonia) and AKI, UTI, VTE including DVT and PE, sepsis, SSI, need for post-operative PCD, LoS, readmissions, 30-day mortality and costs. The authors (MK and MMW) independently judged the quality of the studies using the NOS [22]. Any disagreement was resolved through mutual discussion, and the accuracy of the extracted data was adjudicated further by the senior author (SGB).

### Data synthesis and analysis

The meta-analysis was run through ‘meta’ package in R software, version 4.2.3 (R Core Team, 2023) [23]. Outcomes expressed as median and inter-quartile range were converted to mean and standard deviation for pooled analysis [24]. The pooled effect size of converted versus completed and converted versus open on outcome was estimated using a random-effects model to account for both within-study and between-study variation and provides a more conservative estimate of the overall effect size. RR and associated 95% confidence intervals were calculated for dichotomous data by Mantel–Haenszel models, while SMD and associated 95% confidence intervals were calculated for continuous data using inverse-variance methods by restricted maximum-likelihood estimation process. Statistical heterogeneity among the included studies was assessed using *I*^*2*^, with an *I*^*2*^ of 0–30, 30–60, 50–70, and > 75% representing low, moderate, substantial, and considerable heterogeneity, respectively. The two-sided test was performed for all analyses, 95% confidence interval reported, and level of significance was set at 0.05. Publication bias was assessed using funnel plots and Egger’s test.

## Results

### Baseline demographics

Six retrospective cohort studies (four from the United States [25–28], one from Korea [29] and one multi-national European study [30]) including a total of 20,267 patients, published between 2017 and 2022 were suitable for inclusion (Table 1). While 4 studies [25–28] were registry-based, Lof et al*.*[30] included medium (10–19 MIPD per year) and high (> 0 MIPD per year) volume centres in their study, and Connie et al*. *[29] conducted a single-centre study. The latter [29] included only LPD cases, whilst the other studies [25–28, 30] included both LPD and RPD. Four studies compared all 3 cohorts, namely, (*unplanned*) *converted* MIPD, *successfully completed* MIPD and upfront *open* PD [25, 27–29]. The remaining 2 studies [26, 30] compared only *successfully completed* MIPD to (*unplanned*) *converted* MIPD. Beane et al*. *[25], Hester et al*. *[26] and Lof et al. [30] performed propensity matched analysis between *successfully completed* versus (*unplanned*) *converted* MIPD, whereas Stiles et al*.*[27] performed a propensity matched analysis between (*unplanned*) *converted* MIPD and open PD cohort. For this review, total cohorts were selected from Beane et al*. *[25], Lof et al*. *[30], and Stiles et al*. *[27] with the propensity matched cohorts taken from Hester et al. [26]. Conversion rates ranged between 9.2 and 25.2%, with higher rates being noted in registry-based studies. There were no differences in age or BMI between groups. While Villano et al*. *[28] included patients with malignancy alone, the percentage of patients undergoing PD for malignancy ranged from 55–92% in other studies. There was no significant difference between the groups in terms of the use of neoadjuvant chemotherapy. (*Unplanned*) *converted* MIPD were associated with significantly longer operative duration compared to *successfully completed* MIPD [27] and upfront *open* PD [27, 29]. Conversion was associated with a increased median intraoperative blood loss (500 vs. 275 ml; *p* = 0.005), and rate of blood transfusions (17.2 Vs. 42.2%; *p* < 0.01) [25, 30]. In studies that reported the outcome, conversion did not impact lymph node harvest [29], though patients who underwent an (*unplanned*) *converted* MIPD had lower rates of *R0* resection compared to *successfully completed* MIPD and upfront *open* PD (71.9% vs. 77.8% vs. 77.7% *p* = 0.004) [28] (Table 2).

**Table 1:** Baseline characteristics of the included studies and study populations

Study	n			Conversion rates, %	NOS
	Conv	Comp	Open		
Beane et al., 2017	96	285	5863	25.2	8
Stiles et al., 2018	86	264	86*	24.6	9
Hester et al., 2020	83	83	–	19.7	8
Lof et al., 2021	65	644	–	9.2	8
Connie et al., 2021	33	138	117	19.3	8
Vilano et al., 2022	579	1834	10,011	23.9	9

Conv: Converted, Comp: Completed; NOS: Newcastle Ottawa scale)

**Table 2:** Preoperative and intraoperative characteristics

Characteristics	Conv/ Completed/ Open PD					
	Beane et al., 2017	Stiles et al., 2018	Hester et al., 2020	Lof et al., 2021	Connie et al., 2021	Villano et al., 2022
Age (years)	65 (56–71)/ 65 (58–72)/ 65 (57–73)	–	(64.2 ± 9.9 63.6 ± 10.5)/ –	66 (57–73)/ 68 (63–76)/ –	59.9 ± 12.1/ 65.7 ± 10.9/ 64.1 ± 9.9	66.5 ± 10.4/ 66.7 ± 10.1/ 66.4 ± 10.0
ASA class [n (%) > ASA 2]	213 (74.7)/ 76 (79.2)/ 4453 (75.9)	200(75.8)/ 68 (79)/ –	64(77.1)/ 68(81.9)/ –	123 (20.5)/ 21 (32)/ –	48 (34.8)/ 16 (48.5)/ 57 (48.7)	–
BMI (kg/m^2^)	29.9 (23.0–30.9)/ 27.1 (24.5–31.3)/ 26.5 (23.2–30.2)	–	28.1 ± 5.9/ 27.3 ± 6.1/ –	24 (21–26)/ 24 (22–28)/ –	23.5 ± 2.9/ 23.0 ± 2.5/ 23.3 ± 3.1	–
Pancreatic t exture- Soft n (%)	128 (44.9)/ 26 (27.1)/ 1998 (34.1)	122 (46.2)/ 23 (26.7)/ –	–	–	96(69.6)/ 16 (48.5)/ -	–
MPD < 3 mm, n (%)	81 (28.4)/ 14 (14.6)/ 1360 (23.2)	76 (28.8)/ 11 (12.8)/ –	–	–	3.6 ± 2.2/ 4.8 ± 3.1/ -	–
PC	157 (55)/ 54 (56.3)/ 3247 (55.4)	183 (69.3)/ 60 (69.8)/ –	65 (78.3)/ 67 (80.7)/ –	352 (57.8)/ 45 (69)/ –	88 (63.8)/ 24 (72.7)/ 108 (92.3)	579 (100)/ 1834 (100)/ 10,001 (100)
NAT	61 (21.4)/ 17 (17.7)/ 1326 (22.6)	64 (24.2)/ 16 (18.6)/ –	11 (13.3)/ 10 (12.0)/ –	7 (1.2)/ 2 (4)/ –	3 (2.2)/ 1(3.0)/ 23 (19.7)	141 (24.6)/ 436 (23.8)/ 2,360 (23.6)
Operative duration (mins)	451 (371–559)/ 394 (326–472)/ 355 (277–437)	447 (362–559)/ 391 (326–468)/ 385 (305–447)	473.9 (462)/ 436.6 (428)/ –	420 330–492)/ 415 (339–510)/ –	516.8 ± 96.6/ –/ 449.9 ± 102.9	–
Blood loss (ml)	–	–	–	500 (250–1000)/ 200 (100–400)/ –	645.5 ± 559.4/ –/ 562.1 ± 439.2	–
Peri-operative transfusions	41 (42.7)/ 27 (9.5)/ 1159 (19.8)	–	–	21 (33)/ 77 (12.8)/ –	3 (9.1)/ –/ 15 (12.8)	–
*R0* Resection	–	–	–	49 (80)/ 494 (87.0)/ –	–	–
Vascular Resection	36 (37.5)/ 71 (24.9)/ 1013 (17.3)	–	–	21 (33)/ 29 (4.8)/ –	–	–
Nodal harvest	–	–	–	–	12.4 ± 7.5/ –/13.9 ± 8.9	–

Conv: Converted, Comp: Completed, NOS: Newcastle Ottawa scale, ASA: American Society of Anesthesiologists grade, BMI: Body mass index, MPD: Main Pancreatic duct, PC: Pancreatic cancer, NAT-Neoadjuvant therapy

### Risk of bias and quality assessment

Of the included studies, 2 studies [27, 28] received a NOS score of 9 with the remaining 4 studies [25, 26, 29, 30] scoring 8, suggesting a high quality and low risk of bias for all studies (Table 1).

### Post-operative outcomes (Table 3)

**Table 3:** Post-operative outcomes in individual studies

Outcome	Conv/ Completed/ Open PD, n (%)					
	Beane et al., 2017	Stiles et al., 2018	Hester et al., 2020	Lof et al., 2021	Connie et al., 2021	Villano et al., 2022
30-day Mortality	8 (8.3)/ 8 (2.8)/ 123 (2.1)	4 (4.7)/ 3 (1.1)/ 1 (1.2)	3 (3.6)/ 3 (3.6)/ –	6 (10)/ 18 (3.1)/ –	0/ –/ 0	–
90-day mortality	–	–	–	7 (10.8)/ 26 (4.03)/ –	0/ –/ 0	46 (8)/ 92 (5)/ 611 (6.1)
Overall Morbidity	51(53.1)/ 119 (41.8)/ 2681 (45.7)	63 (73.3)/ 102 (38.6)/ 53 (61.6)	62 (74.7)/ 40 (48.2)/ –	38 (58)/ 359 (58.5)/ –	–	-
Major (≥ CD3) Morbidity	32 (33.3)/ 54 (19.0)/ 1296 (22.1))	–	56 (67.5)/ 31 (37.3)/ –	21 (32)/ 170 (27.7)/ –	–	–
Superficial SSI	8 (8.3)/ 17 (6.0)/ 520 (8.9)	7 (8.1)/ 16 (6.1)/ 11 (12.8)	9 (10.8)/ 3 (3.6)/ –	–	–	–
Deep SSI	1 (1.0)/ 3 (1.1)/ 139 (2.4)		1 (1.2)/ 1 (1.2)/ –	–	–	–
Organ Space SSI	22 (22.9)/ 34 (11.9)/ 791 (13.5)	16 (18.6)/ 27 (10.2)/ 11 (12.8)	17 (20.5)/ 8 (9.6)/ –	–	–	–
Wound dehiscence	2 (2.1)/ 3 (1.1)/ 89 (1.5)	–	2 (2.4)/ 2 (2.4)/ –	–	–	–
PCD placement	29 (30.5)/ 33 (11.7)/ 733 (12.8)	24 (27.9)/ 26 (9.8)/ 11 (12.8)	–	–	–	–
CR-POPF	20 (20.8)/ 34 (12.1)/ 660 (11.4)	26 (30.2)/ 48 (18.2)/ 16 (18.6)	24 (28.9)/ 13 (15.7)/ –	15 (23)/ 135 (22)/ –	5 (15.1)/ –/ 19 (16.2)	–
DGE	19 (20.4)/ 45 (16.0)/ 977 (17.2)	16 (18.6)/ 40 (15.2)/ 17 (19.8)	16 (19.8)/ 15 (18.3)/ –	8 (12)/ 88 (14.5)/ –	7 (21.2)/ –/ 18 (15.4)	–
PPH		–	–	10 (15)/ 60 (9.9)/ –	4 (12.1)/ –/ 1 (0.85)	–
Sepsis	13 (13.5)/ 14 (4.9)/ 827 (9.0)	9 (10.5)/ 13 (4.9)/ 10 (11.6)	–	–	–	–
Re-exploration	10 (10.4)/ 22 (7.7)/ 314 (5.4)	7 (8.1)/ 18 (6.8)/ 5 (5.8)	6 (7.2)/ 6 (7.2)/ –	10 (18)/ 62 (11.5)/ –	3 (9.1)/ –/ 0	–
LoS	8 (7–15)/ 7 (6–10)/ 8 (7–12)	8 (7–12)/ 7 (6–10)/ 9 (6–14)	13.2 ± 9/ 9.8 ± 7/ –	16 (10–24)/ 15 (10–24)/ –	21.7 ± 7.5/ –/ 17.9 ± 8.6	12.2 ± 11.0/ 10.8 ± 8.7/ 11.5 ± 8.8
Re-admissions	14 (14.6)/ 69 (24.2)/ 987 (16.9)	9 (10.5)/ 65 (24.6)/ 21 (24.4)	13 (15.7)/ 14 (16.9)/ –	8 (14)/ 45 (7.5)/ –	4 (12.1)/ –/ 9 (7.7)	55 (9.5)/ 130 (7.1)/ 721 (7.2)

Conv: Converted; Comp: Completed, RR: Risk ratio, SMD: Standardized mean differences; CI: Confidence intervals, CR-POPF: Clinically relevant post operative pancreatic fistula, DGE: Delayed gastric emptying, LoS: Length of stay

#### (Unplanned) converted versus successfully completed MIPD

**Fig. 2:**
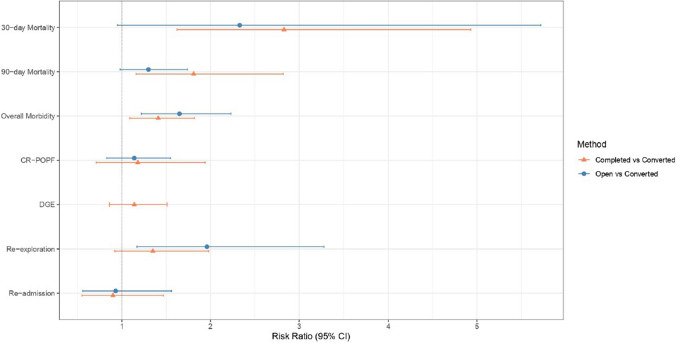
Pooled effect sizes (RR and 95% CI) of outcomes between *successfully completed* MIPD versus (*unplanned) converted* MIPD, and *upfront* open PD versus (*unplanned) converted* MIPD

Beane et al*. *[25] (8.3 vs. 2.8%, *p* = 0.01) and Lof et al.(10 vs. 3.1%, *p* = 0.01) found that 30-day mortality was significantly higher in the (*unplanned*) *converted* group, though Lof et al*. *[30] could not establish any significant association between conversion and mortality in a multivariate logistic regression analysis adjusted for preoperative and intraoperative variables. Two other studies [26, 27] did not find a significant difference in 30-day mortality between the two groups. Pooled analysis (Table 4) showed that the (*unplanned*) *converted* MIPD group had significantly higher 30-day mortality (RR 2.83; CI 1.62; 4.93,* p* = 0.0002, *I*^*2*^ = 0%) in comparison to the *successfully completed* MIPD (Fig. 2***; ****Supplementary Fig. 1a*).

**Table 4:** Percentages, risk ratio and heterogeneity in the outcomes between *successfully completed* MIPD versus (*unplanned) converted* MIPD, and *upfront* open PD versus (*unplanned) converted* MIPD

Outcomes	Converted (Conv) versus Completed (Comp) MIPD							Converted (Conv) versus upfront open PD						
	n	Conv(%)	Comp(%)	RR/ SMD	95% CI	*p*-value	*I*^*2*^, *p*-value	n	Conv(%)	Open(%)	RR/ SMD	95% CI	*p*-value	*I*^*2*^, *p*-value
30-day mortality	4	21 (6.4)	32 (2.5)	2.83	1.62; 4.93	0.0002	0%, 0.56	2	12 (6.6)	124 (2.1)	3.97	2.07; 7.65	< 0.0001	0%, 1.0
90-day mortality	2	53 (8.2)	118 (4.8)	1.81	1.16; 2.82	0.0090	28%, 0.24	–	–	–	–	–	–	–
Overall Morbidity	4	214 (64.8)	620 (48.6)	1.41	1.09; 1.82	0.0087	82, < 0.01	–	–	–	–	–	–	–
CR-POPF	4	74 (22.4)	241 (18.9)	1.18	0.71; 1.94	0.5213	73%, 0.01	3	51 (23.7)	695 (11.5)	1.65	1.22; 2.23	0.0012	0%, 0.40
DGE	4	59 (17.9)	188 (14.7)	1.14	0.86; 1.51	0.3645	0%, 0.87	3	42 (19.5)	1013 (16.7)	1.14	0.83; 1.55	0.4206	0%, 0.77
Re-exploration	4	33 (10)	108 (8.5)	1.35	0.92; 1.98	0.1206	0%, 0.88	3	20 (9.3)	319 (5.3)	1.96	1.17; 3.28	0.011	37%, 0.20
LoS (days)	5	11.41 (82.9)	11.06 (48.0)	0.54	–0.11; 1.18	0.1022	97%, 0.01	4	11.6 (91.1)	10.3 (49.0)	–0.10	–0.65; 0.45	0.7307	93%, 0.01
Re-admission	5	99 (10.9)	323 (10.4)	0.90	0.55; 1.47	0.6740	75%, 0.01	4	82 (10.3)	1738 (10.8)	0.93	0.56; 1.56	0.7970	69%, 0.02

Conv: Converted, Comp: Completed, RR–Risk ratio, SMD: Standardized mean differences, CI: Confidence intervals, CR-POPF: Clinically relevant post-operative pancreatic fistula, DGE: Delayed gastric emptying, LoS: Length of stay

Lof et al*. *[30] noted a significantly higher 90-day mortality in the converted group (13 vs. 4.9%, *p* = 0.012). On pooled analysis, the (*unplanned*) *converted* MIPD group also had significantly higher 90-day mortality (RR 1.81; CI 1.16; 2.82, *p* = 0.009, *I*^*2*^ = 28%) compared to the *successfully completed* MIPD (Fig. 2***; ****Supplementary Fig. 1b*).

In comparison to *successfully completed* MIPD, (*unplanned*) *converted* PD had a significantly higher overall morbidity in studies by Stiles et al*.* (73.3 vs. 38.6%, *p* < 0.001) and Hester et al*.* (74.7 vs. 48.2%, *p* < 0.01). While Hester et al*.* (67.5 vs. 37.3%, *p* < 0.01) also reported significantly higher rates of major (≥ CD3) complications in the (*unplanned*) *converted* cohort, there was no difference in the study by Lof et al*.* (32 vs. 27.7%; *p* = 0.43). Of the 4 studies [25] [26, 27, 30] that compared CR-POPF rates between (*unplanned*) *converted* and *successfully completed* MIPD, significantly higher rates of CR-POPF in the (*unplanned*) *converted* group was reported only by Beane et al*. *[25] (20.8 vs. 12.1%, *p* = 0.02). While no study reported any significant difference in the DGE rates [25] [26, 27, 30] between the 2 groups, Hester et al*. *[26] found higher rates of PPH in the (*unplanned*) *converted* group (43.4 vs. 13.3% *p* < 0.01). Significantly longer hospitalisation was reported by 4 studies [25–28] in the (*unplanned*) *converted* group. Beane et al. [25] reported higher re-exploration rates (10.4 vs. 7.7%, *p* = 0.03) in patients who underwent an (*unplanned*) *conversion*, but there was no difference in the other 3 studies [26, 27, 30]**.** On the contrary, Beane et al*. *[25] found significantly higher readmission rates in patients who underwent a *successfully completed* MIPD (24.2 vs. 14.6%, *p* < 0.01), while there was no difference in the other 4 studies [26–28, 30]**.** Pooled analysis revealed significantly higher overall morbidity (RR 1.41, CI 1.09; 1.82, *p* = 0.0087, *I*^*2*^ = 0.0087) in the (*unplanned*) *conversion* group (Fig. 2***; ****Supplementary Fig. 1c*), while there was no significant difference in CR-POPF, DGE, re-exploration, re-admission or LoS (Table 4) (*Supplementary Fig. 1d–h*).

Significantly higher rates of respiratory complications (pneumonia, need for re-intubation and > 48 h of ventilator dependence) were noted in the (*unplanned*) *conversion* group, while there were no significant differences in renal complications or thromboembolic events [25]**.** Patients in the (*unplanned*) *conversion* group also experienced higher incidence of sepsis including SSIs [25, 26] (*Supplementary Table 2***)**.

##### (Unplanned) converted MIPD versus upfront open PD

Of the 3 studies [25, 27, 29] comparing 30-day mortality between (*unplanned*) *converted* and upfront *open* PD, only Beane et al*. *[25] reported a significantly higher mortality in the former (8.3 vs. 2.1%. *p* = 0.01). Meta-analysis revealed a significantly higher 30-day mortality in the (*unplanned*) *converted* group (RR 3.97, CI 2.07; 7.65, *p* < 0.0001,* I*^*2*^ = 0%) (*Supplementary Fig. 2a*) (Table 4). In the 2 studies [28, 29] comparing 90-day mortality between (*unplanned*) *converted* MIPD and upfront *open* PD, Connie et al*. *[29] had zero event rates, precluding a pooled analysis.

Stiles et al*. *[27] found no difference in overall complications (73.3 vs. 61.6%, *p* = 0.104), and 30-day mortality (4.7 vs. 1.2%, *p* = 0.368) in the (*unplanned*) *conversion* compared to the upfront *open* PD group. Only Beane et al*. *[25] noted a significant increase in CR-POPF in the (*unplanned*) *converted* group (20.8 vs. 11.4%, *p* = 0.02) compared to the upfront *open* PD group. All 3 studies [25, 27, 29] found no difference in rates of DGE. Connie et al*. *[29] reported a significantly higher rate of PPH (12.1 vs. 0.85%, *p* = 0.008), as well as re-exploration rates (*p* = 0.01) in the (*unplanned*) *conversion* group. While Stiles et al*. *[27] found a significantly longer LoS in the upfront *open* group, two other studies reported longer LoS in the (*unplanned*) *conversion* group [25, 28]. Pooled analysis revealed significantly higher rates of CR-POPF (RR 1.65, CI 1.22; 2.23, *p* = 0.0012, *I*^*2*^ = 0%) (*Supplementary Fig. 2b*) and re-exploration (RR 1.96, CI 1.17; 3.28, *p* = 0.011, *I*^*2*^ = 37%) (*Supplementary Fig. 2d*), while there was no difference in DGE and re-admission rates and LoS (*Supplementary Fig. 2c, e and f*) (Table 4). Connie et al*. *[29] reported significantly higher costs per patient in the (*unplanned*) *conversion* group in comparison to upfront *open* PD [$21,886.4 ± 10,594.4 vs. $17,168.9 ± 4,973.1; *p* = 0.018). Assessment using funnel plots and Egger’s test did not reveal significant publication bias in any of the outcomes except LoS (*Supplementary Figs. 3a–h and 4a–f*).

### Predictors of conversion

Male sex [26–30], advanced age [11, 26–29], ASA grade III-IV [29, 30], smoking [27] and patients who reported recent weight loss [27] had a higher likelihood of unplanned conversion. Hard pancreatic texture was found to be associated with (*unplanned*) *conversion* in 2 studies [27, 29]. Another factor found to be more prevalent in patients undergoing conversions was the presence of wider pancreatic ducts [29]. Tumours > 4 cm [30], pancreato-biliary primary [30], and those requiring vascular [27] [26, 30] and/or multi-visceral [26, 30] resection were more likely to necessitate (*unplanned*) intraoperative *conversion*. (*Unplanned*) *conversions* were also significantly more common following LPD compared to RPD [26–28, 30]. Medium volume centres (10–19 MIPD annually) had a higher (*unplanned*) *conversion* rate compared to high volume (> 20 MIPD annually) (15.2 vs. 4.1%, *p* < 0.001) [30]. Only two studies [29, 30] reported indications for intra-operative conversion (Table 5**).**

**Table 5:** Indications for unplanned conversion during MIPD

Indications for intra-operative conversion	n (%)
Vascular involvement	28 (28)
Adhesions	20 (20)
Bleeding	13 (13)
Technical difficulties	9 (9)
Oncologic concerns	7 (7)
Obesity	6 (6)
Pancreatitis	5 (5)
Small pancreatic duct	2 (2)
Concomitant colonic resection	2 (2)
High pCO_2_	2 (2)
Unknown	5 (5)

pCO2–partial pressure of carbon dioxide

## Discussion

These data demonstrate that (*unplanned*) *converted* MIPD were associated with an increased 30- and 90-day mortality and overall morbidity compared to *successfully completed* MIPD. Patients undergoing (*unplanned*) *converted* MIPD experienced significantly higher 30-day mortality, CR-POPF and re-exploration rates compared upfront *open* PD.

Observational, case-matched studies on MIPD [31] [32] [33] demonstrate longer operative times, but less operative blood loss and shorter hospitalization in LPD with complication rates and oncological outcomes comparable to OPD. However, registry-based studies [34, 35] have advised caution owing to increased mortality rates after LPD, especially in low-volume centres. The four RCTs [4–6, 9] published to date comparing LPD vs. OPD have been unable to demonstrate a clear indication of post-operative morbidity and mortality, as they were likely underpowered to detect these differences. The conversion rates in these trials ranged from 3 to 25%. Unfortunately, since converted patients were predominantly analysed in the laparoscopic group on an “intention-to-treat” basis, the true implications of an intra-operative conversion were not readily evident. The meta-analysis by Zhang et al*. *[36] comparing LPD vs. open PD highlighted the advantages of LPD in terms of lesser intra-operative blood loss, higher *R0* resection rates and lymph node yield, lower perioperative overall morbidity, and shorter length of hospitalization. No difference in survival was noted. In keeping with the IDEAL framework for surgical innovation, all novel interventions should preferably be evaluated against the current standard in a randomized controlled trial (RCT) [37]. And so, an updated meta-analysis of RCTs comparing LPD vs. open PD confirmed a significantly lower blood loss and surgical site infection rate in the LPD cohort, while the approaches were similar with respect to other outcomes [38]. The benefits of MIPD in terms of improvements in optics, surgical instrumentation, and increased access to training [12, 39] have led to an increased interest amongst surgeons to attempt MIPD.

This study presents the most updated appraisal of the literature on the impact of unplanned intra-operative conversions in MIPD acknowledging that they constitute an inherent problem in the learning curve of minimally-invasive surgery. Interestingly, there were no significant differences in the rates of pancreas-specific complications (CR-POPF, DGE), re-explorations rates, or readmissions rates. Though less frequent in comparison to LPD, *unplanned conversions* in RPD had more significant consequences, which may be attributed to the longer duration in RPD to actually convert to an open procedure with consequently greater blood loss. It could also reflect a problem of selection bias wherein only the most difficult procedures were converted in RPD, whereas LPD procedures were converted more easily [40]. The significantly higher 30-day mortality, CR-POPF and re-exploration rates in the *unplanned conversion* MIPD cohort compared to the upfront *open* PD cohort is also intriguing bearing in mind that the data in the present study is from retrospective series, wherein patients for MIPD would have been following a strict selection policy.

Though broadly defined into three phases [41], that is, competency, proficiency, and mastery, there exists little standardization in literature on what constitutes an established definition of a rigorous learning curve required to perform a surgery as complex as MIPD. Criticism of the literature on learning curve studies point out that they are derived from CUSUM analyses [42] based on intra-operative parameters such as operative duration and/ or intra-operative blood loss rather than postoperative outcomes such as complication rates or LoS. Additionally, there exists a significant correlation between study sample sizes and number of procedures needed to surpass the learning curve, questioning the meaningfulness and applicability of these results [43]. It is paramount that future studies investigating the subject take into account not only surgeon (previous surgical [44] and simulation [45] experience, procedure-specific training and clinical fellowships [46]) and patient (BMI [47], comorbidities and tumour factors [10]) characteristics, but institutional expertise as well, which includes annual procedural volumes and team familiarity [48].

Patient selection appears to be of paramount importance in MIPD, given the risks associated with unplanned conversions. The current review highlights patient (elderly, male, smokers, ASA III/ IV, recent history of weight loss), pancreatic gland, and tumour (> 4 cm, pancreato-biliary tumours) characteristics associated with higher risk of intra-operative conversion, that need to be factored in while selecting patients for MIPD. The Miami guidelines state that trainees should have passed the learning curve for open PD (> 60) before undertaking training in MIPD. Centres should be performing at least 50 PD annually in addition to minimum annual volume of 20 MIPDs [1]. This may be operationalized by devising strict national and international surgical society guidelines. Further, utilization of risk prediction scores like Difficulty scoring system (DSS)[49] or PD-ROBOSCORE [50] will likely aid better patient selection for MIPD.

The study is not without limitations. The definition of conversion varied between centres/ studies. Categorization of conversion into elective and emergency in future studies may facilitate rational comparison of outcomes. Secondly, studies might have had surgeons at different stages of learning curve, and a uniform definition of learning curve might enable assessment of the impact of surgeon experience and centre volume on the risk of conversion. Finally, we combined LPD and RPD together due to non-availability of stratified data, though it is obvious that both the approaches differ not only in terms of impact of conversion, but risk factors as well.

As surgical innovations become more complex and the burden of age and comorbidities in the surgical patient population continues to increase, understanding the benefits and risks associated with surgical interventions becomes ever more important. We need to move beyond the traditional endpoints of mortality and resource use towards more pertinent measures of morbidity, patient-reported outcomes, and functional status. At the present time, the implementation of MIPD must be guided by an appreciation of surgeon training and (also institutional) capability and optimum patient selection, as *unplanned conversions* are fraught with the attendant risk of morbidity and mortality.

## Supplementary Information

Below is the link to the electronic supplementary material.

Supplementary file1 (DOCX 1970 kb)

## Abbreviation

AKI: Acute kidney injury
ASA: American Society of Anaesthesiology
BMI: Body mass index
CD: Clavien-Dindo
CR-POPF: Clinically-relevant post-operative pancreatic fistula
DGE: Delayed gastric emptying
DVT: Deep vein thrombosis
LPD: Laparoscopic pancreatoduodenectomy
MeSH: Medical Education Subject Headings
MIPD: Minimally-invasive pancreatoduodenectomy
NOS: Newcastle–Ottawa scale
PCD: Percutaneous drainage
PD: Pancreatoduodenectomy
PE: Pulmonary embolism
PRISMA: Preferred Reporting Items for Systematic Reviews and Meta-analyses
PPAP: Post-pancreatectomy acute pancreatitis
PPH: Post-pancreatectomy haemorrhage
RPD: Robotic pancreatoduodenectomy
RR: Risk ratio
SMD: Standardised mean differences
SSI: Surgical site infections
UTI: Urinary tract infection
VTE: Venous thromboembolism
