# Supplementary material for: Impact of Unplanned Intra-Operative Conversions on Outcomes in Minimally Invasive Pancreatoduodenectomy
Source: World J Surg. 2023 Jul 12;47(10):2507–18. doi: 10.1007/s00268-023-07114-1 (PMC10473988; doi:10.1007/s00268-023-07114-1)

**Supplementary tables and figures**

**Supplementary Table 1: Search Strategy**

| **Search** | **Search terms** | **Number of results** |
| --- | --- | --- |
| **PubMed PubMed** | | |
| **#1** | “Conversion” OR “Open conversion” OR “Intra-operative conversion” OR “Unplanned Conversion” | 278,198 |
| **#2** | “Pancreatectomy” OR “Pancreaticoduodenectomy” OR “Pancreatic resection” OR “Minimally invasive pancreaticoduodenectomy” OR “Laparoscopic pancreaticoduodenectomy” OR “Robotic Pancreaticoduodenectomy” OR “Hybrid pancreaticoduodenectomy” | 50,383 |
| **#3** | “Pancreatic Cancer” OR “Pancreatic Carcinoma” OR “Pancreatic adenocarcinoma” OR “Pancreatic Tumour” | 139,923 |
| **#4** | # 1 AND # 2 AND # 3 | 607 |
| **Google Scholar** | | |
| **#1** | “Conversion” OR “Open conversion” OR “Intra-operative conversion” OR “Unplanned Conversion” | 5,160,000 |
| **#2** | “Pancreatectomy” OR “Pancreaticoduodenectomy” OR “Pancreatic resection” OR “Minimally invasive pancreaticoduodenectomy” OR “Laparoscopic pancreaticoduodenectomy” OR “Robotic Pancreaticoduodenectomy” OR “Hybrid pancreaticoduodenectomy” | 23,600 |
| **#3** | “Pancreatic Cancer” OR “Pancreatic Carcinoma” OR “Pancreatic adenocarcinoma” OR “Pancreatic Tumour” | 2,070,000 |
| **#4** | #1 AND #2 AND #3 | 5,180 |
| **Cochrane Cochrane Library** | | |
| **#1** | “Conversion” OR “Open conversion” OR “Intra-operative conversion” OR “Unplanned Conversion” | 1867 |
| **#2** | “Pancreatectomy” OR “Pancreaticoduodenectomy” OR “Pancreatic resection” OR “Minimally invasive pancreaticoduodenectomy” OR “Laparoscopic pancreaticoduodenectomy” OR “Robotic Pancreaticoduodenectomy” OR “Hybrid pancreaticoduodenectomy” | 123 |
| **#3** | “Pancreatic Cancer” OR “Pancreatic Carcinoma” OR “Pancreatic adenocarcinoma” OR “Pancreatic Tumour” | 68 |
| **#4** | #1 AND #2 AND #3 | 26 |

**Supplementary Table 2: Non-surgical morbidity**

| **Outcome** | | **Conv/ Completed/ Open PD, n (%),** | | | | | |
| --- | --- | --- | --- | --- | --- | --- | --- |
|  |  | **Beane et al, 2017** | **Stiles et al,**  **2018** | **Hester et al, 2020** | **Lof *et al*,**  **2021** | **Connie et al, 2021** | **Villano et al, 2022** |
| Cardiac | MI | 0 (0)/  2 (0.7)/  65 (1.1) | 0 (0)/  2 (0.8)/  0 (0) | - | - | - | - |
|  | Cardiac arrest | 4 (4.2)/  6 (2.1)/  63 (1.1) | 3 (3.5)/  3 (1.1)/  1 (1.2) | - | - | - | - |
| Thromboembolic | DVT | 6 (6.3)/  6 (2.1)/  143 (2.4) | 5 (5.8)/  6 (2.3)/  4 (4.7) | - | - | - | - |
|  | PE | 1 (1.0)/  5 (1.8)/  71 (1.2) | - | 1 (1.2)/  1 (1.2)/  - | - | - | - |
| Pulmonary | Pneumonia | 7 (7.3)/  4 (1.4)/  245 (4.2) | 6 (7.0)/  4 (1.5)/  5 (5.8) | 5 (6.0)/  2 (2.4)/  - | - | - | - |
|  | Re-Intubation | 9 (9.4)/  14 (4.9)/  241 (4.1) | 7 (8.1)/  11 (4.2)/  5 (5.8) | 6 (7.2)/  4 (4.8)/  - | - | - | - |
|  | Vent dependence >48 hrs | 10 (10.4)/  11 (3.9)/  179 (3.1) | 6 (7.0)/  9 (3.4)/  1 (1.2) | 6 (7.2)/  3 (3.6)/  - | - | - | - |
| Urinary | UTI | 4 (4.2)/  10 (3.5)/  195 (3.3) | 3 (3.5)/  12 (4.5)/  7 (8.1) | 3 (3.6)/  2 (2.4)/  - | - | - | - |
|  | AKI | 3 (3.1)/  4 (1.4)/  94 (1.6) | 1 (1.2)/  2 (0.8)/  0 (0.0) | - | - | - | - |

**(Abbreviations-** Conv- Converted; Comp- Completed; MI- Myocardial infarction; DVT- Deep vein thrombosis; PE- Pulmonary Thromboembolism; UTI- Urinary tract infection; AKI- Acute kidney injury)

**Supplementary Figure 1: Forest plots comparing outcomes between *(unplanned) conversion* and *successfully completed* MIPD: a) 30-day mortality, b) 90-day mortality, c) Overall morbidity, d) CR-POPF, e) DGE, f) Re-exploration rates, g) Re-admission rates, and h) Length of stay (LoS)**

**
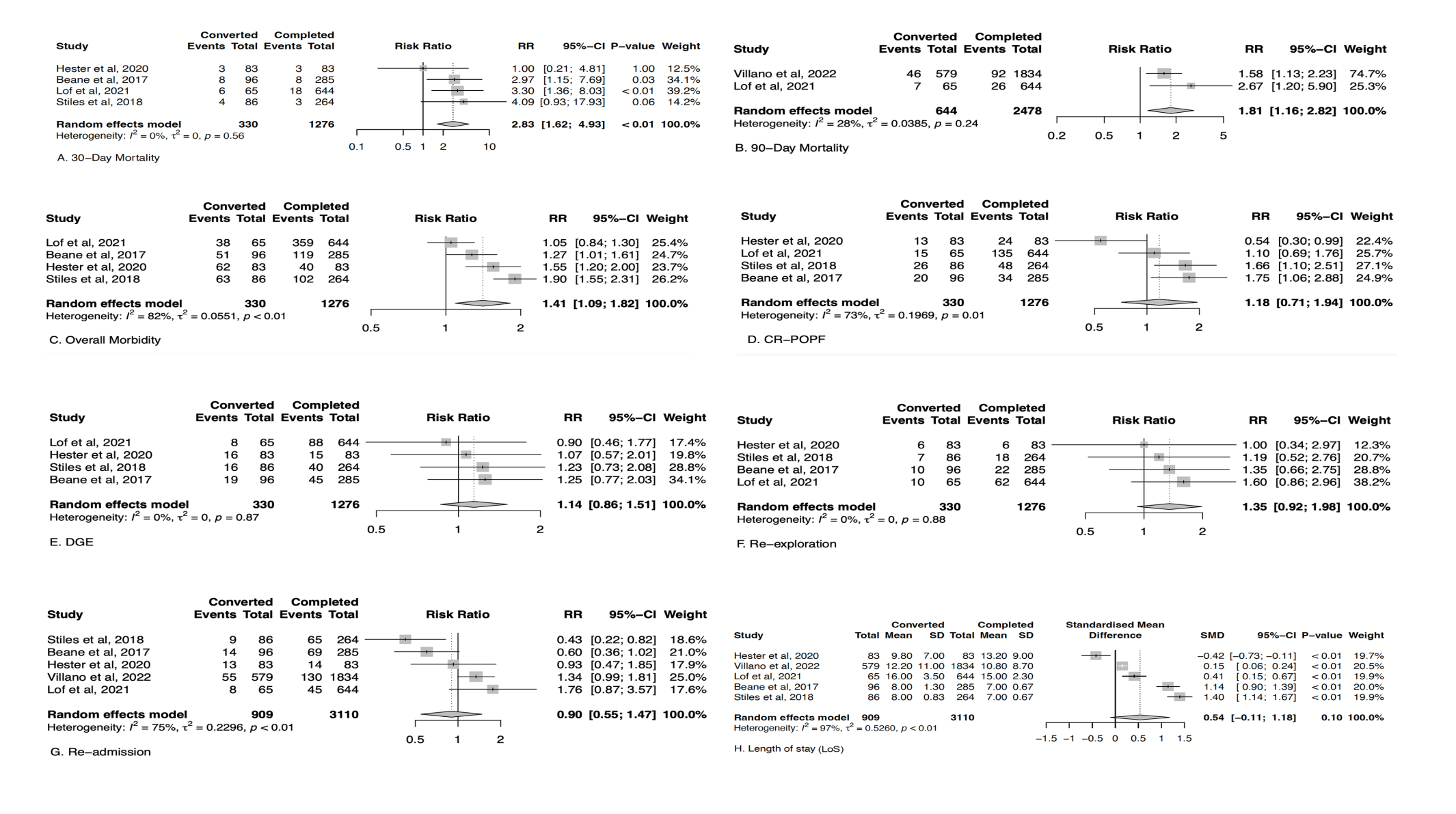
**

**Supplementary Figure 2: Forest plots comparing outcomes between *(unplanned) converted MIPD* and upfront *open* PD: a) 30-day mortality, b) CR-POPF, c) DGE, d) Re-exploration rates, e) Re-admission rates, and f) Length of stay (LoS)**

**
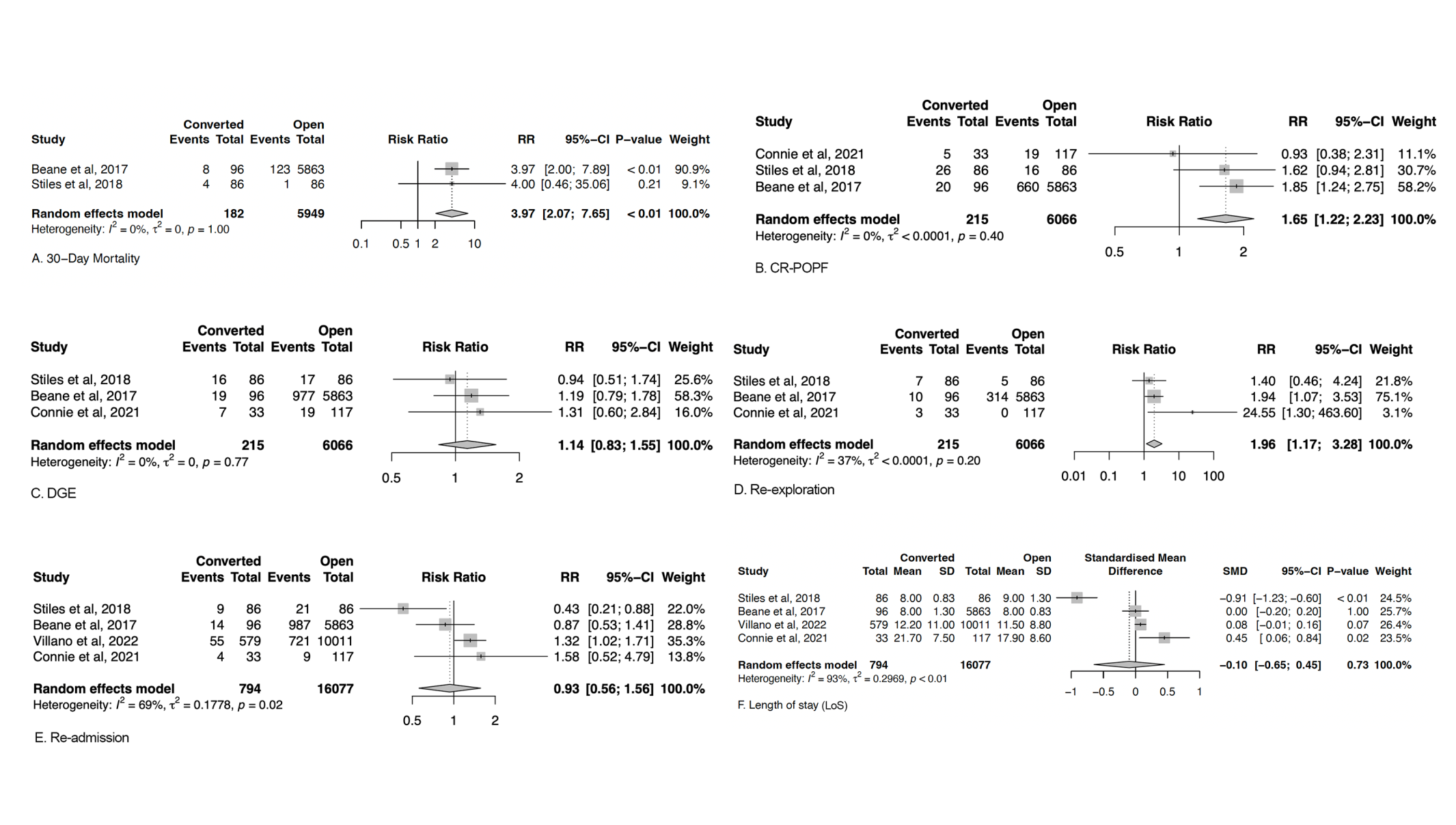
**

**Supplementary figure 3: Funnel plot comparing outcomes between *(unplanned) conversion* and *successfully completed* MIPD: a) 30-day mortality, b) 90-day mortality, c) Overall morbidity, d) CR-POPF, e) DGE, f) Re-exploration rates, g) Re-admission rates, and h) Length of stay (LoS)**

**
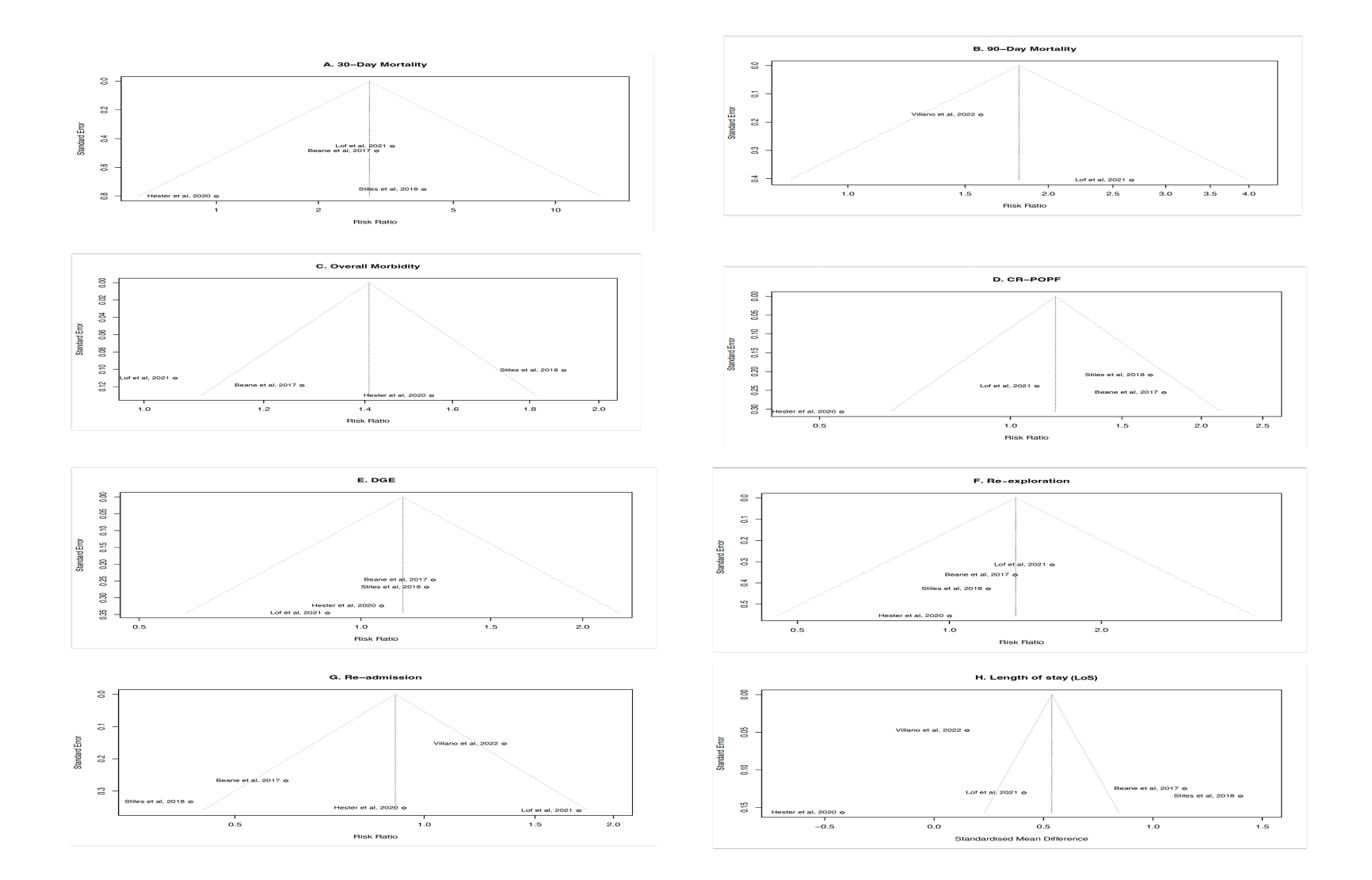
**

**Supplementary figure 4: Funnel plot comparing outcomes between *(unplanned) converted MIPD* and upfront *open* PD: a) 30-day mortality, b) CR-POPF, c) DGE, d) Re-exploration rates, e) Re-admission rates, and f) Length of stay (LoS)**


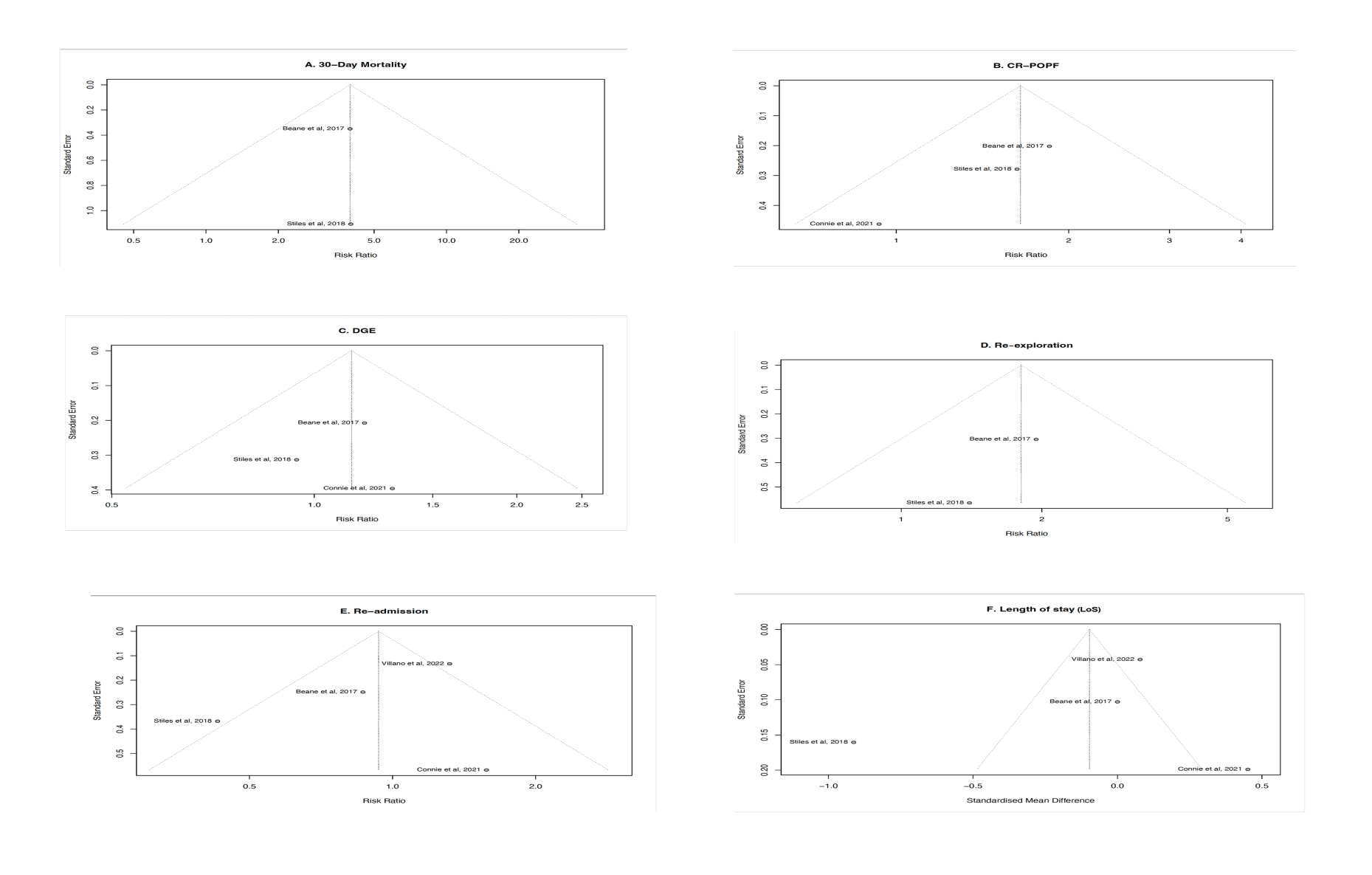

Supplement: Supplementary file 1 — Supplementary file1 (DOCX 1970 kb) [file 268_2023_7114_MOESM1_ESM.docx]
